# Examining How African American Family Dementia Caregivers Conceptualize and Manage Crisis Events

**DOI:** 10.1093/geroni/igaa057.2778

**Published:** 2020-12-16

**Authors:** Quinton Cotton, Laura Block, Jennifer Morgan, Andrea Gilmore-Bykovskyi

**Affiliations:** 1 University of Wisconsin-Madison, Madison, Wisconsin, United States; 2 University of Wisconsin-Madison School of Nursing, Madison, Wisconsin, United States; 3 Georgia State University, Atlanta, Georgia, United States

## Abstract

African American (AA) family dementia caregivers report high unmet needs, which often culminate in crisis – an unplanned stressful situation requiring immediate decision. However, perspectives from AA caregivers regarding crisis are lacking. To gain insight into caregivers’ conceptualization and experiences of crisis, we used community/coalitional-based recruitment of AA caregivers to conduct semi-structured interviews with 34 AA caregivers which were analyzed using thematic analysis (N=34, 94% female, 56% ages 65 to 74). AA caregivers largely perceived crisis as stressful events, a normal part of caregiving and viewed management of these events as routine. Crisis was characterized as ongoing, lengthy or emergent, sometimes necessitating external support (.e.g. hospitalization). Caregivers managed crisis by increasing caregiving work, de-prioritizing their own health and needs, involving family and friends, and accessing emotional support through neighborhood connections. These perspectives can inform future culturally-tailored interventions that are responsive to AA strengths, values, and help seeking preferences.

